# Strain Gauges Based on CVD Graphene Layers and Exfoliated Graphene Nanoplatelets with Enhanced Reproducibility and Scalability for Large Quantities

**DOI:** 10.3390/s17122937

**Published:** 2017-12-18

**Authors:** Volkan Yokaribas, Stefan Wagner, Daniel S. Schneider, Philipp Friebertshäuser, Max C. Lemme, Claus-Peter Fritzen

**Affiliations:** 1 Department of Mechanical Engineering, University of Siegen, 57076 Siegen, Germany; volkan.yokaribas@uni-siegen.de (V.Y.); philipp.friebertshaeuser@student.uni-siegen.de (P.F.); 2 Chair for Electronic Devices, RWTH Aachen University, 52074 Aachen, Germany; wagner@amica.rwth-aachen.de (S.W.); lemme@amo.de (M.C.L.); 3 Advanced Microelectronic Center Aachen (AMICA), AMO GmbH, 52074 Aachen, Germany; schneider@amo.de; 4 ZESS (Center of Sensor Systems), University of Siegen, 57076 Siegen, Germany

**Keywords:** CVD graphene 1, graphene nanoplatelets 2, strain gauge 3, piezoresistive effect 4, reproducibility 5, spray deposition 6, static load test 7

## Abstract

The two-dimensional material graphene promises a broad variety of sensing activities. Based on its low weight and high versatility, the sensor density can significantly be increased on a structure, which can improve reliability and reduce fluctuation in damage detection strategies such as structural health monitoring (SHM). Moreover; it initializes the basis of structure–sensor fusion towards self-sensing structures. Strain gauges are extensively used sensors in scientific and industrial applications. In this work, sensing in small strain fields (from −0.1% up to 0.1%) with regard to structural dynamics of a mechanical structure is presented with sensitivities comparable to bulk materials by measuring the inherent piezoresistive effect of graphene grown by chemical vapor deposition (CVD) with a very high aspect ratio of approximately 4.86 × 10^8^. It is demonstrated that the increasing number of graphene layers with CVD graphene plays a key role in reproducible strain gauge application since defects of individual layers may become less important in the current path. This may lead to a more stable response and, thus, resulting in a lower scattering.. Further results demonstrate the piezoresistive effect in a network consisting of liquid exfoliated graphene nanoplatelets (GNP), which result in even higher strain sensitivity and reproducibility. A model-assisted approach provides the main parameters to find an optimum of sensitivity and reproducibility of GNP films. The fabricated GNP strain gauges show a minimal deviation in PRE effect with a GF of approximately 5.6 and predict a linear electromechanical behaviour up to 1% strain. Spray deposition is used to develop a low-cost and scalable manufacturing process for GNP strain gauges. In this context, the challenge of reproducible and reliable manufacturing and operating must be overcome. The developed sensors exhibit strain gauges by considering the significant importance of reproducible sensor performances and open the path for graphene strain gauges for potential usages in science and industry.

## 1. Introduction

The two-dimensional (2D) material graphene has exceptional intrinsic properties, including a very high mobility [1], extraordinary mechanical strength [2] with a stretchability of over 20% [3]. Over the last decades, serious efforts have been conducted to synthesize high quality graphene by different methods. Epitaxial growth [4], chemical vapor deposition (CVD) [5] and exfoliation methods [6,7] are the most common fabrication methods. To expand the range of potential applications for graphene-based sensors the use for strain gauge application is assessed. Metallic strain gauges [8,9], semiconductor strain gauges [10] or fiber optic strain sensors [11] have been arranged in various mechanical systems for aerospace, civil and mechanical engineering applications. Operational and environmental conditions outside a controlled laboratory setting require the quantification of multiple application scenarios. Therefore, the functionality of such sensors is severely limited [12].

Many recent experimental and numerical investigations have been devoted to the piezoresistive effect (PRE) and to achieve high gauge factors (GF) with graphene strain gauges [13,14,15]. A summary by Zhao et al. distinguishes between different mechanisms for the PRE [16]. Beside the intrinsic resistance change due to structure deformation of a graphene layer [17,18,19], the change of contact resistance of adjacent graphene sheets plays a prevalent role. The resistivity changes of adjacent graphene sheets can be determined by changing their overlapped area [20] and tunneling distance [14,21,22]. Breakage of conductive paths in an electrical network embedded with graphene sheets, which is initialized by high density of cracks formation due to deformation, lead also in resistance changes [15,23]. A detailed overview of various strain gauges based on graphene with their corresponding sensing mechanism in one-dimensional, two-dimensional and three-dimensional architectures are summarized in [24]. Most of the findings focus on tuning sensor performance to achieve the highest sensitivity [25,26] by tailoring sensors’ microstructure [25] or fabrication method [22]. Nevertheless, there is still a distribution in the PRE after manufacturing which is an exclusion criteria for the sensor. It is of significant importance that the sensors reproducibility and repeatability are given for large quantities. Therefore, aspects such as simple manufacturing and easy application as well as reliable operating principle of graphene strain gauges need to be assessed for industrial application. Here, we investigate the intrinsic PRE for CVD graphene and the PRE of exfoliated GNP networks by considering the requirements of reproducible strain gauge performances. In particular, the latter provides an efficient large-area transfer to the desired substrates by e.g., inkjet-printing [27,28] or spray deposition [29], with low production cost and the ability to tailor the geometry according to its application. The comparatively complex manufacturing and transfer process of CVD graphene with its susceptibility to defects [30] may be the limitations for utilizing graphene layers for high volume strain gauge production. Nevertheless, an approach for enhanced stability of the PRE shall be considered by increasing the number of layers (mono-, bi- and multiple layer/multi-layer (6–8 sheets)), which has not been investigated so far. In this way, very thin and transparent strain sensitive sensors are developed for application scenarios such as wearable sensors on human skins [23] and microelectromechanical systems (MEMS) [31]. Furthermore, the application of GNP strain gauges is investigated by developing a reproducible sensor in order to overcome the stability requirements for potential usages in scientific and industrial applications. 

## 2. Materials and Methods 

### 2.1. CVD Grown Graphene for Strain Gauge Application

CVD can be used to grow mono- or multiple-layers of graphene on a catalytic transition metal substrate like copper (Cu) [5] or nickel (Ni) [32], respectively. The Cu substrate is first annealed at 1000 °C in an atmosphere of hydrogen and argon to remove the oxide on the surface. A hydrocarbon gas, like methane (CH_4_) is used which dissociates on the Cu substrate into carbon atoms forming the graphene lattice. Graphene islands with different lattice orientations are created all over the substrate which expand and grow together [33]. A grain boundary is formed when the graphene islands merge. These may influence the electronic properties (e.g., electron mobility through scattering effect at the grain boundaries [34,35]) or the mechanical properties increasing the probability of crack formation [35,36]. The growth process on a Cu substrate is self-limiting to a mono-layer of graphene. The grain size can be defined by adjusting the growth parameters [33] between several micrometers [34] up to millimeter size [5]. Besides mono-layer, mechanically stacked bi-layer was created and 6 to 8 layers graphene on a Cu-foil were purchased from ACS Materials [37]. These three CVD graphene samples need to be transferred from the growth to the device substrate using a standard wet transfer process [34].

#### 2.1.1. Fabrication Steps of CVD Graphene Strain Gauges

**Figure 1:**
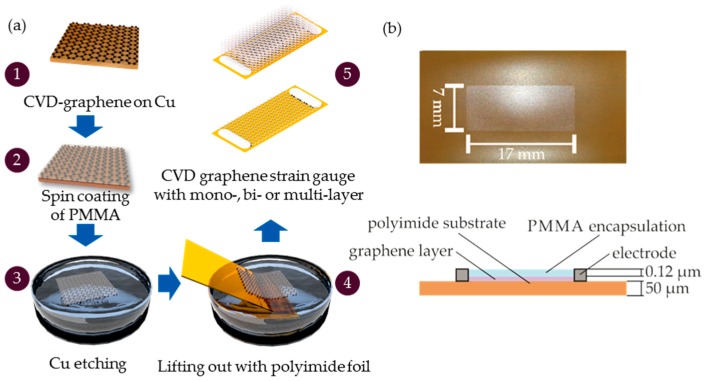
(**a**) Schematic description of the main fabrication steps of mono-, bi- and multi-layer CVD graphene: (1) CVD graphene on Cu foil, (2) spin coating of polymethyl methacrylate (PMMA), (3) Cu etching, (4) lifting out of graphene-PMMA stack with a PI foil, (5) contacting by using silver paste; (**b**) fabricated strain gauge on PI substrate.

Polyimide (PI) is a common substrate used for metallic strain gauges and other flexible electrical devices. The material is a flexible polymer which has a maximal elongation of 20% and a minimal bending radius of 0.3 mm as well as high heat resistance and temperature stability over a range of −269 °C to 400 °C [38]. The graphene transfer from the Cu substrate to the PI is a critical fabrication step. A standard wet transfer method was used for the strain gauge shown in Figure 1. 

Polymethyl methacrylate (PMMA) (E-beam resist ARP 679.02 from Allresist) is spin-coated onto mono- and multi-layer graphene on Cu for 20 s at a rotational speed of 1200 rpm, followed by a hot bake step to evaporate the solvent. The Cu foil is placed in a sodium persulfate solution to etch the Cu. The PMMA-graphene stack is lifted out of the liquid using the PI substrate with a thickness of 50 µm. The graphene layer is blow dried using nitrogen gas and to promote the adhesion to the substrate. In terms of mechanically stacked bi-layer graphene the fabrication steps are modified. The PMMA-graphene stack is lifted out with a second mono-layer of graphene on Cu. A thermal treatment in an oven at 150–180 °C removes the liquid and brings the two layers in close contact [39]. The Cu etching process is repeated and the stacked graphene bi-layer is transferred onto a PI foil in the last process step. The PMMA layer is not removed and serves as an encapsulation to increase the sensors stability. 

#### 2.1.2. Characterization of CVD Graphene Layers 

**Figure 2:**
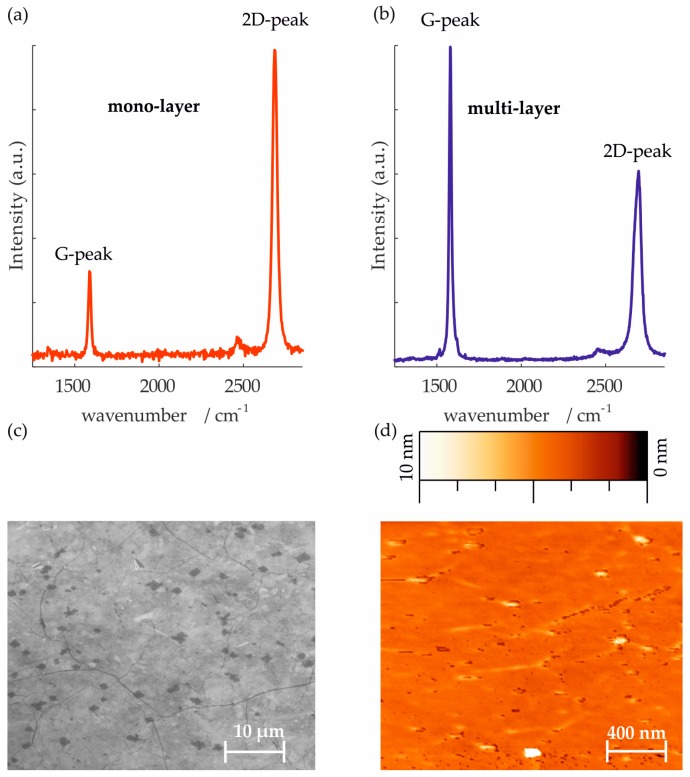
Representative Raman spectra with an excitation wavelength of 532 nm measured on CVD grown graphene: (**a**) mono-layer graphene with 2D/G intensity ratio of 3.45; (**b**) multi-layer graphene (6 to 8 layers) with 2D/G intensity ratio of 0.6 purchased from ACS Materials; (**c**) SEM image displaying structure of our mono-layer graphene on a Si-SiO_2_ wafer; (**d**) High-resolution AFM measurement displaying roughness of a mono-layer graphene on a Si-SiO_2_ wafer.

The mono-layer graphene used for the devices was grown in-house in a Moorfield NanoCVD rapid thermal processing cold wall reactor system on a 2 × 2 cm^2^ Cu sheet [40]. The structure and the roughness of our mono-layer graphene were determined by scanning electron microscope (SEM) and atomic force microscopy (AFM) (see Figure 2). 

Raman spectroscopy is the most commonly used method to qualitatively analyze graphene [41]. Properties, like the quality of graphene and the number of layers can be determined by quantifying the peak intensity, position or full width half maximum (fwhm). A typical Raman spectrum shows three distinct peaks for graphene. The first peak, called the D peak, is located at about 1350 cm^−1^ and corresponds to defects within the sp^2^ hybridized honeycomb structure of graphene [42]. The next peak in a graphene is the G peak, found at about 1580 cm^−1^ [41]. By calculating the intensity ratio of the G peak and the third peak, which is called 2D peak, located at about 2790 cm^−1^, the number of graphene layers can be determined [42]. A spectrum of a nearly perfect graphene mono-layer shows a 2D/G peak intensity ratio greater than 2 and decreases with increasing number of layers [41,43]. 

The Raman spectrum of the used mono-layer as well as multi-layer graphene for the strain gauge application show the characteristic spectrum with a very low D peak intensity which is negligible in comparison to the other peaks, indicating high quality graphene with few defects (see Figure 2). The 2D/G ratio is roughly four for the mono-layer and lower than 1 for the multiple layer [41]. The Raman spectra were measured with a WITec alpha 300R system and a 532 nm laser for excitation. 

### 2.2. Liquid Exfoliated Graphene Nano Platelets (GNP) for Strain Gauge Application

Apart from the described bottom up manufacturing approach in Section 2.1 a top-down process can be used to gain graphene from bulk graphite. In comparison to the strong covalent intersheet bonds in graphite, weak van der Waals forces act as intralayer bonds between the graphite [44]. In the liquid exfoliation process shear forces are induced through sonication or shear mixing to break up these bonds in an aqueous solution by adding surfactants [45]. This results in GNP suspensions. The uniformity and flake size is limited, but larger quantities of low-cost graphene can be achieved with liquid exfoliation [7,45]. 

#### 2.2.1. Fabrication Steps of GNP Strain Gauges

**Figure 3:**
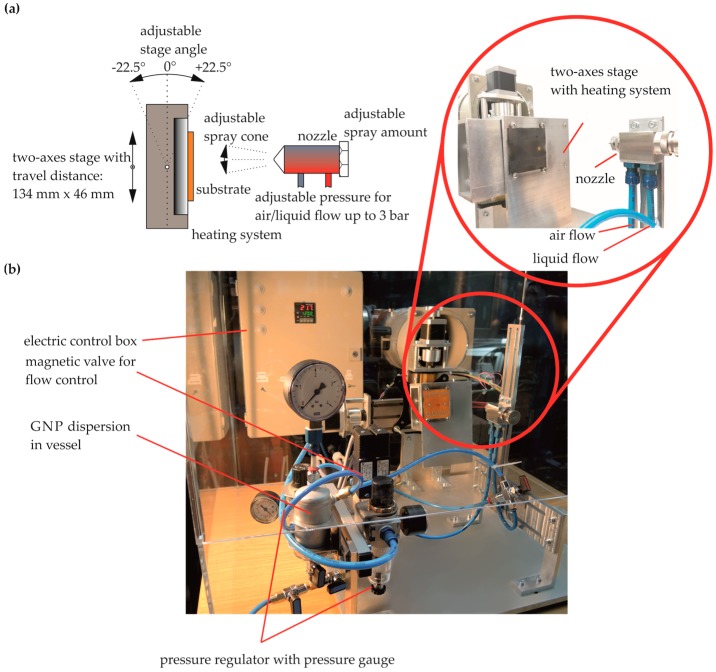
(**a**) Schematically shown spray deposition; (**b**) spray deposition device of aqueous dispersion with GNP [49].

**Figure A1:**
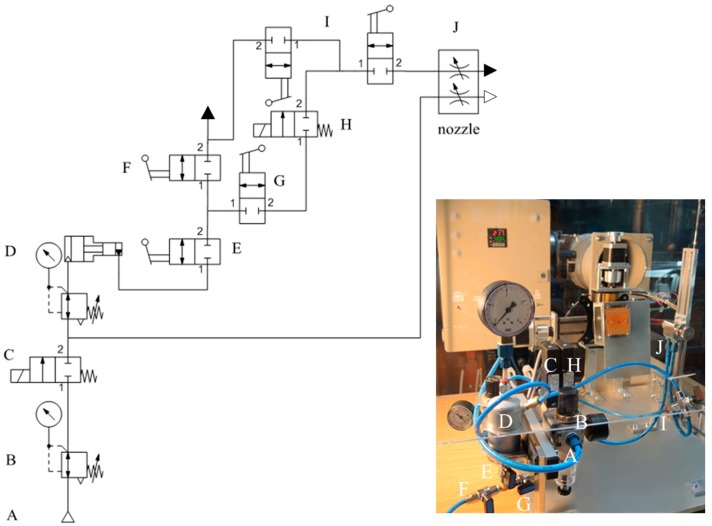
Pneumatic/Hydro plan of the spray deposition system for GNP strain gauges fabrication (A: compressed air supply, B: pressure regulator, C: magnetic valve for air flow controlling, D: vessel for dispersion, E: manual valve, F: manual valve to remove the dispersion from the vessel, G: manual vessel for liquid supply, H: magnetic valve for liquid flow controlling, I: distribution valve, J: nozzle).

To utilize exfoliated GNP in dispersion, drop casting [46], vacuum filtration [47], Inkjet printing [27] or spray deposition [48] techniques can be applied. Particularly, the implementation of GNP by spray deposition provides large-area application without a changing in the structural properties (stiffness, weight, etc.) of the underlying structure. Highly scalable and automated fabrications of GNP films require adjustable process parameters, like a motorized stage with stepper motor and the amount of dispersion to ensure a high level of operational reliability and accuracy (see Figure 3). A key role for the performance of spray deposition is seen in the nozzle (Figure 3a). The flow rate is limited by an annular gap which is adjustable through the position of a needle within the two-substance nozzle. We fabricated the GNP strain gauges with a 0.2 mm nozzle and an atomization air pressure of 2 bar. An optimum between the spray parameters (e.g., flow rate, pressure of atomization air and liquid, spray distance,) need to be found in future work. Figure 3b describes a spray deposition system which was developed for strain gauge manufacturing. A detailed pneumatic/hydro plan of the spray deposition device is shown in the Appendix (Figure A1).

**Figure 4:**
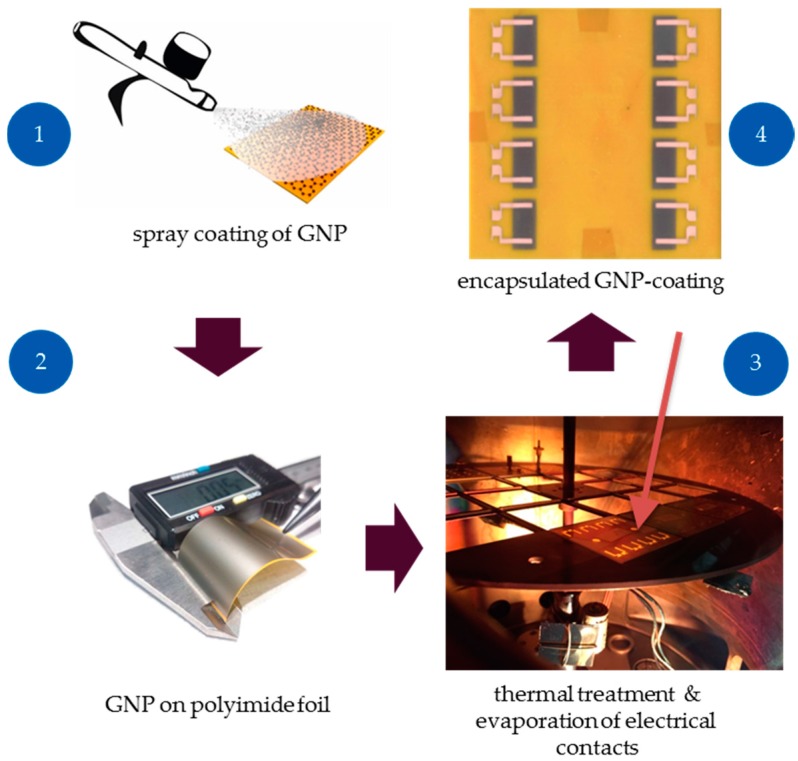
Strain gauge fabrication steps with GNP: spray deposition of GNP (1), GNP on flexible polyimide foil (2), thermal treatment and evaporation of electrical contacts (3), GNP strain gauges after encapsulation (4) [49].

After spray deposition, ready-made GNP strain gauges on a 50 µm thick polyimide foil were fabricated. The main fabrication steps are described in Figure 4. We observed an improved attachment to the polymer substrate after annealing in an oven of the GNP film at 300 °C for 30 min. Functional groups in the polymeric chain possibly interact with the surface and form electrostatic bonds [50]. Moreover, evaporation of solvents may enhance the adhesion since the GNP are in closer contact with the substrate. Metal contacts were deposited on polyimide substrate by thermal evaporation with a shadow mask. Firstly, chromium is applied as adhesion promotion layer with a thickness of 30 nm followed by 500 nm copper to connect the electrical wires by soldering. A spin-coated and cured 10 µm thick polyimide layer serves as an encapsulation. It provides a good processability and adhesion with the GNP as well as the coated substrate. The encapsulation significantly improves the sensors durability, as no removal of the GNP can occur due to contact abrasion. Moreover, an encapsulation compensates the cross-sensitivity to humidity, which is shown in a short-time test in [49].

#### 2.2.2. Characterization of Liquid Exfoliated GNP 

**Figure A2:**
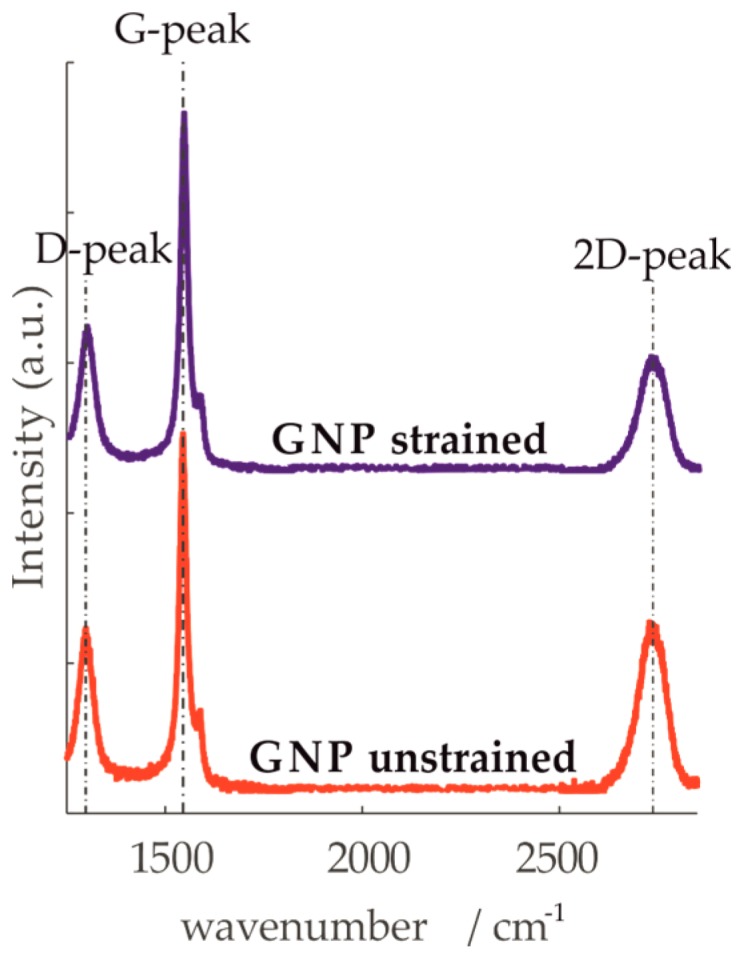
Representative Raman spectra with an excitation wavelength of 532 nm measured for unstrained (with 2D/G intensity ratio of 0.32) and strained (1%) state of a GNP film.

The aqueous dispersion used for the strain gauge devices (purchased from Thomas Swan) are shear exfoliated GNP with a sheet resistance of approximately 20 Ω/□ for a film of 25 µm thickness [45]. The concentration of GNP within the aqueous dispersion is 1 mg/mL. Sodium cholate (NaC) was used as an anionic surfactant with a concentration of 0.28% in order to prevent agglomeration and to keep the individual graphene platelets separated from each other [45]. Transmission electron microscopy (TEM) measurements showed GNP sizes in the range of 300–800 nm with an average thicknesses of less than 10 layers [45]. The presence of graphene in the aqueous dispersion is confirmed by the Raman spectroscopy (Appendix Figure A2). In comparison to the Raman spectrum of CVD grown graphene the Raman spectrum of liquid-phase exfoliated graphene shows a higher D peak intensity. Shear exfoliation reduces the size of the nanosheets by increasing the number of particles simultaneously. As a consequence the edge contributions of each nanosheet will be higher and may result in the distinct D peak [41].

#### 2.2.3. Modeling Procedure 

The conductance behavior of connected clusters in a randomly ordered network may be described by the percolation theory [51]. This approach is often associated with physical models in material science. Applying this theory to a network of randomly positioned particles, it is assumed that the network becomes electrically conductive for a critical volume fraction of connected GNP, which is also known as a percolation threshold [52].

After formation of the percolated network, the working principle of the PRE in a network can be generally explained by the tunneling effect [21], which considers two effects: the conductivity changes of adjacent graphene sheets due to deformation by changing their overlapped area [20] and tunneling distance [14,21,22]. In contrast to CVD graphene the GNP do not deform during deformation of the substrate due to slippage effects between the GNP [53]. The comparison of the Raman spectra for strained and unstrained states of GNP is shown in the Appendix (Figure A2). 

Simplifying the GNP to circular rigid bodies, the motion of the particles can be estimated. For a low-voltage range the tunneling resistivity is given by Simmon’s equation [21] as:

(1) $$\rho_{tunneling}=\frac{h^{2}}{e^{2}\sqrt{2m\lambda }}\exp\left(\frac{4\pi d}{h}\sqrt{2m\lambda }\right),$$

Here, *e* is the elementary charge, *m* is the mass of an electron and *h* is the Planck constant. The tunneling distance *d* is assumed to be the smallest possible distance between the nanoparticles by considering the lower limit of van der Waals distance between adjacent graphene sheets [54]. λ is the potential barrier of the insulator through which the tunneling mechanism occurs.

**Figure 5:**
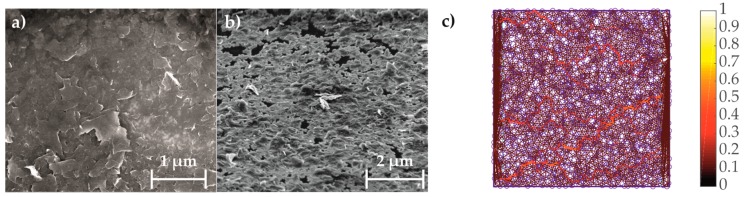
(**a**) SEM image of GNP coating (top view); (**b**) SEM image of GNP coating (52° inclined view); (**c**) numerical 2D-model with high density of randomly distributed GNP and the main conductive paths (highlighted in yellow-red) [49].

A laminated morphology of the GNP coating can be observed in the scanning electron microscope (SEM) image in Figure 5. Derived from the surface morphology of the GNP coating, a numerical 2D-model with hardcore-softshell GNP is developed, which are randomly distributed in a rectangular coated area for a representative element (see Figure 5). As the volume fraction is high enough, GNP are involved in forming conductive paths from the left to the right electrode. Due to changes of position and orientation of GNP, the paths within the GNP network changes and the PRE occurs. The proposed model is similar to 2D-models in the literature [55] and shall predict the sensing behavior of the proposed GNP strain gauge. 

### 2.3. Characterization and Experimental Testing Method for Electromechanical Behaviour 

The resistance change can be measured by a Wheatstone bridge circuit (WBC) with a high resolution for small strain fields. The GF was calculated by considering the offset in a quarter bridge of the WBC after application on a mechanical structure. The strain gauge sensor was attached to the structure via an adhesive layer. A simple cantilever beam setup (length 300 mm and width 30 mm) is a low-cost device for sensitivity tests with a well-defined strain field. For data acquisition the amplifier module Quantum MX840B of HBM is used which provided an internal transducer for the WBC. 

The relative change in the resistance ΔR/R of a conductor consists of two terms: a change due to the alteration in the conductor geometry by strain ε and transverse contraction ν, and an alteration in electrical resistivity ρ due to changes in electron mobility, which is expressed by the PRE Δρ/ρ:

(2) $$\frac{\Delta R}{R}=\varepsilon (1+2\nu )+\frac{\Delta \rho }{\rho },$$

The *GF* is a general measure for the sensitivity of the sensor and quantifies the relative change of the resistance *R* due to the applied strain *ε* on graphene by

(3) $$GF=\frac{1}{\varepsilon }\frac{\Delta R}{R},$$

Mono-, bi- and multi-layer CVD graphene as well as GNP strain gauges were investigated in a quasi-static load test with a strain field from −0.1% up to 0.1%, where their sensitivities were quantified. 

## 3. Results and Discussion

### 3.1. Experimental Testings of CVD Graphene 

#### 3.1.1. Quasi-Static Load Test

**Figure 6:**
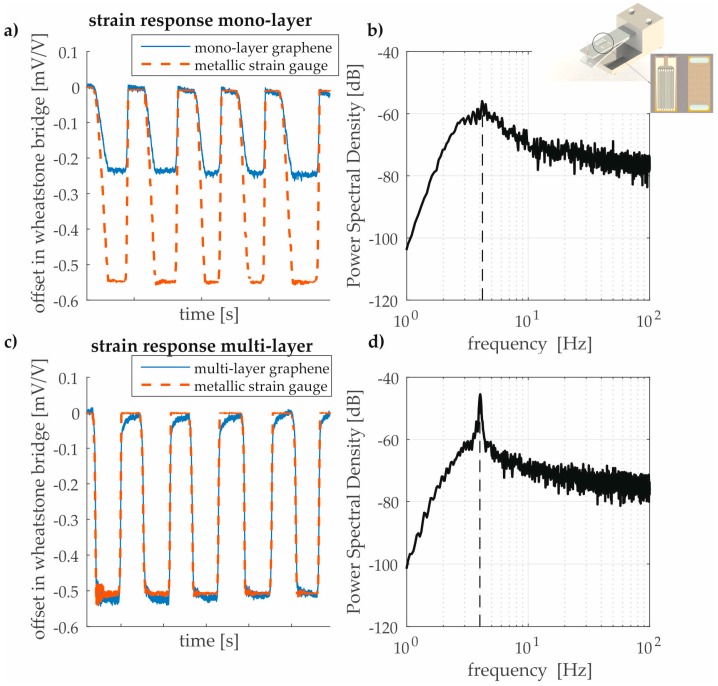
(**a**,**c**) Electromechanical responses of mono- and multi-layer CVD graphene in comparison to metallic strain gauges for five load cycles; (**b**,**d**) determination of the first eigenfrequency during load test (using a cut-off frequency filter for frequencies lower than 3 Hz).

The results presented in Figure 6 show that 2D materials are suitable for structural strain sensing on metallic surfaces in macro-scale in an explanatory manner. The results of the quasi-static tests for CVD graphene are comparable to strain gauges made by bulk materials (e.g., GF of a constantan measuring grid is approximately two [8]). It can be seen from a comparison with the metallic strain gauge (dashed line) that the graphene sensors follow the swelling loads. In the case of multi-graphene viscoelastic effects can be observed. By applying the loads, the cantilever beam induces free mechanical vibration due to elastic effects. The CVD graphene also enables the detection of the first eigenfrequency during the free vibration of the cantilever. The structural analysis of the first eigenmode is shown in a power spectral density spectrum for mono- and multi-layer graphene by using a cut-off frequency filter for frequencies lower than 3 Hz. The higher the sensitivity of the sensor, the higher the distinct peak for the first eigenfrequency. In the case of mono-layer graphene, the peak is still visible but the signal is overlapped with the pre-dominant noise level. Quantification of the sensors performance in dynamic behavior requires more effort and research. Within the present work the quasi-static behavior shall be solely considered as mentioned above. 

#### 3.1.2. Piezoresistive Effect 

The reliability and reproducibility are the key points in the sensor application. Therefore, the sensitivity for mono-, bi- and multi-layer graphene were investigated of a rectangular shaped CVD graphene sensor with an aspect ratio of approximately 4.86 × 10^8^, which is the highest measured so far. 

**Figure 7:**
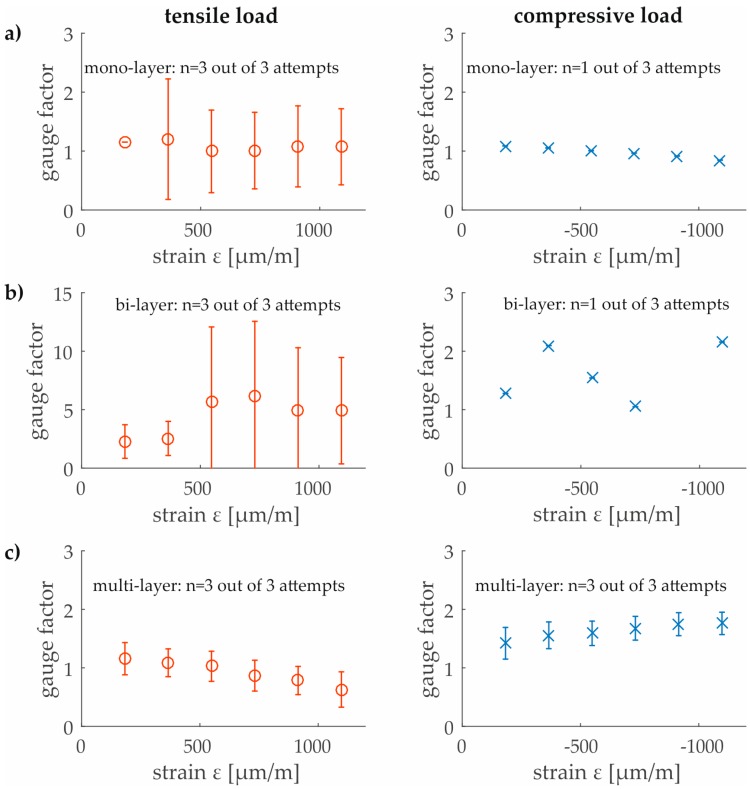
Gauge factors (including number of measurements, the mean and the standard deviation) of mono- (**a**), bi- (**b**) and multi-layer (**c**) CVD graphene for tensile and compressive loads on the cantilever beam.

Using the cantilever setup and applying compression and tension loads, a strain field from around −0.1% up to 0.1% can be partially detected by the graphene sensors. In case of mono- and bi-layer graphene the tensile loads were detectable while the compressive loads are unreliable in two of three attempts. The scattering in both cases is higher in comparison to the multi-layer graphene. The GF of the bi-layer graphene are the most strongly scattered. Nevertheless, it can be seen that only the multi-layered graphene layer provided appropriate measuring signals for all applied sensors. Moreover, the multi-layer graphene shows a contrasting behavior in comparison to the mono-layer sensors. By increasing the strain, a more sensitive electromechanical behavior to a compressive load than to a tensile load is observed (see Figure 7).

#### 3.1.3. Discussion of the Results 

In a CVD graphene based strain sensor, a large number of mechanisms of action occur which cause the piezoresistive effect (PRE). The intrinsic PRE of a graphene membrane is different to the network of GNP and shall be considered in Section 3.2. As a result of the deformation, the carrier mobility and carrier density are changed in case of a CVD graphene [56]. It is assumed that either dilation or contraction changes the area of the unit cell and modifies the relative orientation of atoms. Nearest neighbor hopping length of electron changes which leads to changes in hopping amplitude [57]. Smith et al. found that the crystallographic orientation of graphene is independent of the direction of applied strain and therefore an isotropic PRE can be assumed [57]. The assumption that even small mechanical deformations of the crystal lattice cause a band gap in the electron band structure has been refuted experimentally and theoretically [56]. The velocity of electrons in graphene is described with the Fermi velocity v_F_. Investigations of Huang et al. with nanoindentation of graphene layer with a width of 1.5–4 μm show that the normalized resistance change is related to the changes in the Fermi-velocity [56]. Smith et al. also confirm that the resistivity increases by increasing the strain since the Fermi-velocity decreases [57]. The proposed results of CVD mono-layer graphene lie within the same value range as predicted by other authors. Huang et al. determined a GF of approximately 1.9 in strain gauges [56]. Investigations of Smith et al. revealed a GF with maximum of 4.33 and an average value of 2.92 in pressure sensors [31]. 

In our investigations with a very high aspect ratio (approximately 4.86 × 10^8^) of the fabricated CVD graphene strain gauges, a higher scattering of the GF was observed in the bi-layer in comparison to the mono- and multi-layer strain gauges. In this context, the presence of defects can play a pre-dominant role as they can induce significant changes in the drift mobility, which may result in a higher variation of the GF [35]. These defects can occur in form of holes, wrinkles, buckling and can be caused during the fabrication, transferring and operating process [33,58,59]. Besides that, the influence of the carrier substrate is also not negligible. The type of carrier substrate [60] and its surface roughness affects both the conductivity and the quality of the graphene layer [61]. It is also assumed that van der Waals bonds are the pre-dominant adhesion bindings between graphene and the carrier substrate [54]. However, since this type of binding is very weak, it is conceivable that relative displacement occurs. Zhu et al. distinguish between standing collapsed wrinkles and folded wrinkles in the morphology of graphene [58]. When a certain height is exceeded, the standing fold collapses and forms a three-layered area with the underlying graphene layer. Thus, changes of the electromechanical behavior in form of scattering of GF as well as offsets in the signal during quasi-static test may also be explained.

Nevertheless, in contrast to bi-layer graphene, defects have less influence on the electromechanical behavior for multi-layer graphene, since defects of individual layers may become less important in the current path. This may lead to a more stable response and, thus, resulting in a lower scattering in the intrinsic PRE for the two dimensional graphene layers with a very high aspect ratio. 

### 3.2. Experimental Testings of GNP 

#### 3.2.1. Quasi-Static Load Test

**Figure 8:**
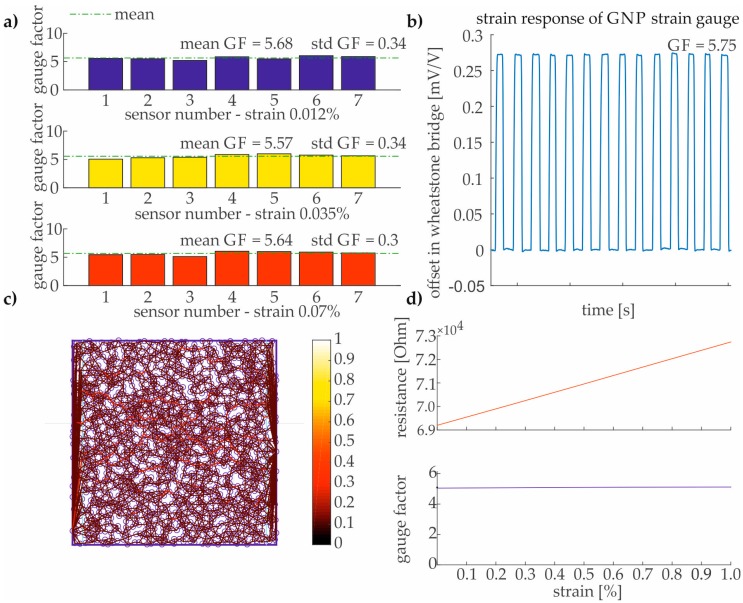
(**a**) Comparison of GF for seven strain gauges from the same production batch (data statistics mean and standard deviation (std)); (**b**) exemplary electromechanical response of a GNP strain gauge (sensor number seven); (**c**) representative element of the coating with a GNP concentration of Vc = 40%; (**d**) electromechanical behavior of the numerical 2D-model for the representative element far beyond the percolation threshold [49].

The results in Figure 8 show that the GNP strain gauges are suitable for structural strain sensing. The applied strain ε is systematically increased up to 0.12%, which is identical to the CVD graphene strain sensor measurements. A GF between 5.5 and 6 could be determined by using the aforementioned measurement setup (see Figure 8a,b) and the spray deposition technique for fabricating GNP strain gauges. These results are at least 2.5 times higher than strain gauges made from CVD graphene (see Section 3.1). The previously proposed numerical 2D-model (see Section 2.2.3) is in good agreement with the experimental results for the used aqueous dispersion (Figure 8c,d). The theoretical estimation of a high-volume fraction (Vc) with densely packed GNP due to dissipation of water after thermal annealing represents the tailored initial conditions of the model far beyond the percolation threshold. The potential barrier of the GNP network in the used aqueous dispersion is considered low since a matrix material (such as e.g., polymer) does not exist in the fabricated strain gauge. Moreover, the simulation of strain up to 1% (Figure 8d) predicts a linear increase of the resistance which contributes to a reproducible sensor behavior over an extended strain range.

#### 3.2.2. Numerical Investigations of Piezoresistive Effect

**Figure 9:**
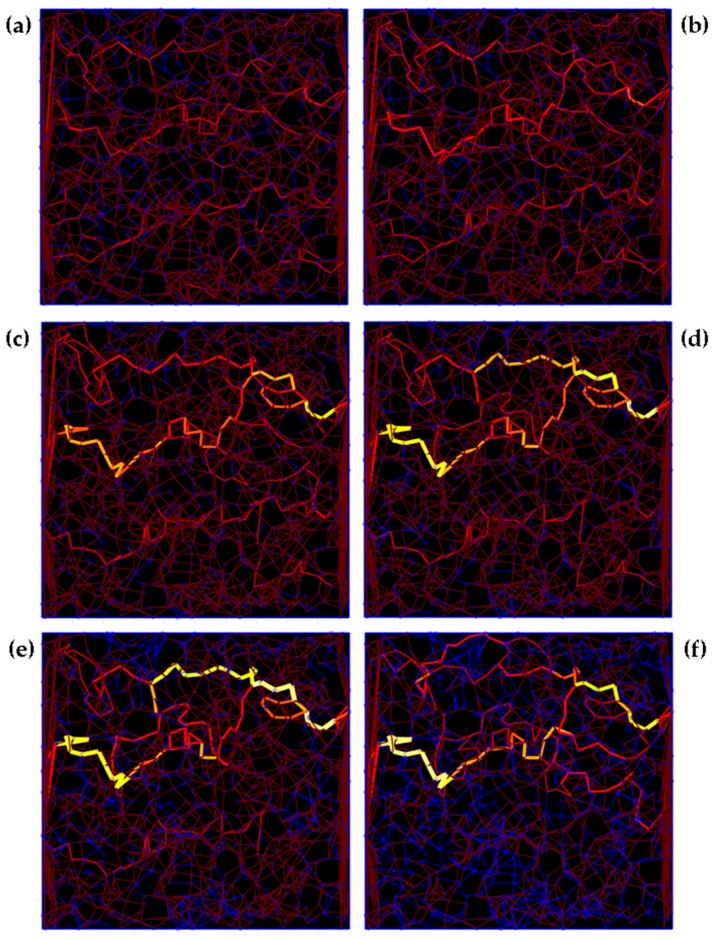
Changes in the current paths (from left to right) within the identical rectangular network by increasing the potential barrier: λ = [0.04 (**a**); 0.23 (**b**); 0.95 (**c**); 2.14 (**d**); 3.81 (**e**); 8.57 (**f**)] eV.

Many recent investigations with theoretical models were performed to understand the conductivity and PRE in a nanocomposite enhanced with conductive nanoparticles, like GNP [20,55] or carbon nanotubes [62]. To ensure reproducibility in a strain gauge, a minimal deviation of the GF is proposed, which is seen in the experimental results in Section 3.2.1. A model-assisted analysis of the network with GNP within a strain gauge was qualitatively investigated. The prevalent effect in a network of GNP is the tunneling effect. For this purpose, the influence of the potential barrier λ shall be investigated in the network of GNP. The conductive paths have been generated by the model. By increasing the potential barrier for a given network the current density for the path changes, which is represented by the line width of the individual paths (see Figure 9). The thicker the current path inside the GNP network, the higher the current density of this path. Figure 9a–f show that for small values of λ = 0.04 eV and λ = 0.23 eV the current flow is nearly homogenously distributed within the network. However, with increasing barrier, single conductive paths are playing a more dominant role within the network while other paths minimize their contribution in the current flow. This effect is particularly shown in the last network with λ = 8.57 eV, where only few critical current paths exist.

In a model-assisted parameter study, the reproducibility of the gauge factor and the initial resistance as a function of the potential barrier is statistically quantified for 30 attempts. The number of particles inside the network is set to a fixed value. Table 1 shows that the standard deviation (std) of the GF and the initial resistance increase by increasing the potential barrier. As a result of the reduction of the effective particles, which have a contribution to the current paths of the network, the reproducibility of the GNP strain gauge decreases. In this case uncertainties in fabrication process also (e.g., deviation of volume fraction, distribution of GNP) strongly affect the sensors reproducibility. Therefore, there is still a conflict between objectives in sensitivity and reproducibility. 

**Figure 10:**
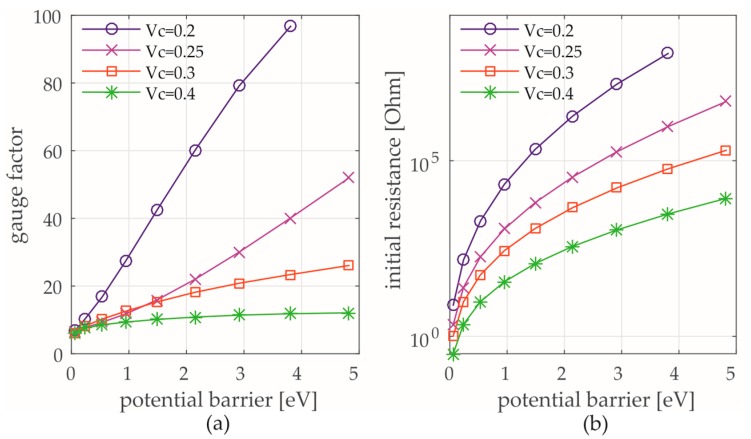
GF (**a**) and the initial resistance (**b**) as a function of the potential barrier.

Furthermore, the GF and initial resistance are determined as a function of the potential barrier λ with a simultaneous increase of the volume fraction Vc (see Figure 10a,b). From the lower limit to the upper limit of λ = 5 eV, the GF increases for a Vc of 0.4 by approximately 40%, for Vc = 0.3 by 150%, for Vc = 0.25 by 460% and for Vc = 0.2 by 470%. Since the mean tunneling distances in the network increases with decreasing Vc, the sensitivity (GF) becomes higher. For very small potential barriers of λ < 0.5 eV, the GF becomes nearly independent of the Vc. In this case, decreasing Vc is not an appropriate method to increase the GF. The mean initial tunneling distance between the particles has no greater influence on the sensitivity by considering such a condition. Thus, it seems obvious that the used GNP film has a potential barrier of λ < 1 eV.

#### 3.2.3. Discussion of the Results 

It can be concluded that higher potential barriers emphasize a higher sensitivity and adjustability but a lower reproducibility at the same time in the case of randomly oriented networks. For very small potential barriers, the sensitivity decreases strongly. A higher sensitive strain sensor can only be achieved by using a sufficiently high potential barrier. In this way, the PRE is strongly determined by the change of tunneling distances between adjacent graphene sheets. In addition, the GF can be adjusted by the Vc over a large value ranges. In the case of fabricated strain gauges with aqueous dispersion, the results confirm a high reproducibility with a GF of approximately 5.6. By considering a high-volume fraction (which represents the highly dense packed state of the GNP network for our strain gauge) and a low potential barrier, the electromechanical behavior of the spray deposited strain gauge is verified. As a consequence, uncertainties in fabrication of spray coatings (e.g., deviation of the amount of GNP, thickness of GNP, random distribution) can be overcome by a low potential barrier. Thus, our fabricated GNP strain sensors are mainly focused on enhanced reproducibility and scalability for large quantities with a low variation on the sensing behaviour for the initial state. The long-term durability shall be considered in fatigue tests for future studies.

## 4. Conclusions

In the context of sensor development, we have investigated the inherent piezoresistive effect of individual CVD graphene layers as well as the electromechanical network behavior of GNP by considering the requirement of reproducibility, which is one of the key points in sensor applications. 

Using a simple cantilever, we were able to characterize the PRE in a well-defined strain field. In case of CVD graphene, we achieved a higher reproducibility by increasing the number of layer. Thus, defects have less influence on the electromechanical behavior, since defects of individual layers may become less important in the current path. This led to a more stable response and, thus, resulting in a lower scattering of the electromechanical behavior. 

Minimal deviation of the GF over a batch of fabricated GNP strain gauges is achieved far beyond the percolation threshold for densely packed networks. These experimental measurements were confirmed in a model-assisted approach for the used GNP dispersion. Further investigations may deal with the conflict of objectives between sensitivity and reproducibility.

This work provides the path for development of strain gauges based on graphene. Very thin and transparent strain sensitive sensors may be developed for application scenarios such as wearable sensors on human skins or microelectromechanical systems (MEMS). The implementation of integrated GNP coatings by spray deposition allows a large area sensing system and creates new options for smart structures with sprayable sensors. Future work will be extended from quasi-static to dynamic load tests by considering the environmental cross-sensitivities. 

## Figures and Tables

**Table 1:** Reproducibility of the GNP network as a function of the potential barrier.

Potential Barrier (eV)	Std GF (%)	Std Initial Resistance (%)
0.04	6.2%	4.1%
0.24	9.9%	8.0%
0.95	22.0%	17.6%
2.14	25.1%	20.8%
3.81	37.0%	39.4%
8.57	101.8%	66.6%
